# Differential impact of metabolic syndrome on subclinical atherosclerosis according to the presence of diabetes

**DOI:** 10.1186/1475-2840-12-41

**Published:** 2013-03-04

**Authors:** Ki-Bum Won, Hyuk-Jae Chang, Hyeon-Chang Kim, Kyewon Jeon, Hancheol Lee, Sanghoon Shin, In-Jeong Cho, Sung-Ha Park, Sang-Hak Lee, Yangsoo Jang

**Affiliations:** 1 Department of Cardiology, Myongji Cardiovascular Center, Kwandong University College of Medicine, Goyang, Republic of Korea; 2 Department of Cardiology, Yonsei Cardiovascular Center, Yonsei University College of Medicine, Seoul, Republic of Korea; 3 Department of Preventive Medicine, Yonsei University College of Medicine, Seoul, Republic of Korea; 4 Severance Biomedical Science Institute, Seoul, Republic of Korea; 5 Present address: Yonsei Cardiovascular Center, Yonsei University College of Medicine, 50 Yonsei-ro, Seodaemun-gu, Seoul 120-752, Republic of Korea

**Keywords:** Metabolic syndrome, Diabetes, Atherosclerosis

## Abstract

**Background:**

Metabolic syndrome (MS) is associated with increased risks of diabetes and atherosclerotic cardiovascular disease. However, data on the impact of MS and its individual components on subclinical atherosclerosis (SCA) according to diabetes status are scarce.

**Methods:**

Surrogate markers of SCA, brachial–ankle pulse wave velocity (baPWV), and carotid intima–medial thickness (IMT) and plaque were assessed in 2,560 subjects (60 ± 8 years, 33% men) who participated in baseline health examinations for a community-based cohort study.

**Results:**

The participants included 2,149 non-diabetics (84%) and 411 diabetics (16%); 667 non-diabetics (31%) and 285 diabetics (69%) had MS, respectively. Diabetics had significantly higher baPWV and carotid IMT, and more plaques than non-diabetics (p < 0.001, respectively). Individuals with MS had significantly higher baPWV and carotid IMT than those without MS only among non-diabetics (p < 0.001, respectively). Among MS components, increased blood pressure was significantly associated with the exacerbation of all SCA markers in non-diabetics. The number of MS components was significantly correlated with both baPWV and carotid IMT in non-diabetics (baPWV: r = 0.302, p < 0.001; carotid IMT: r = 0.217, p < 0.001). Multiple regression showed both MS and diabetes were significantly associated with baPWV (p < 0.001, respectively), carotid IMT (MS: p < 0.001; diabetes: p = 0.005), and the presence of plaque (MS: p = 0.041; diabetes: p = 0.002).

**Conclusions:**

MS has an incremental impact on SCA in conditions without diabetes. The identification of MS and its individual components is more important for the risk stratification of CVD in non-diabetic individuals.

## Background

Metabolic syndrome (MS) represents a clustering of several cardiovascular (CV) risk factors including abdominal obesity, impaired glucose intolerance, dyslipidemia, and hypertension, with insulin resistance as a major characteristic [1,2]. It was recently estimated that MS is common, affecting 24% of adults in the US and 11–19% in Korea [3,4]. MS is associated with the development of coronary heart disease (CHD) and stroke [5,6].

MS has been promoted as a means of identifying the risk of diabetes development. A number of different definitions of MS include diabetes as part of the diagnostic criteria of MS. Recently, it has been strongly recommended that conditions with established diabetes or cardiovascular disease (CVD) should be excluded from the definition of MS, because MS is a pre-morbid condition rather than a clinical diagnosis [7]. However, there is a paucity of data supporting this recommendation, especially regarding atherosclerosis. In addition, the effects of MS and its individual components on subclinical atherosclerosis (SCA) according to diabetes status are unknown.

The intima–medial thickness (IMT) and plaque of the carotid artery can be measured noninvasively using high-resolution B-mode ultrasound. Brachial–ankle pulse wave velocity (baPWV) is used as a reproducible index of arterial elasticity and stiffness. Both increased baPWV and thickened IMT of the carotid artery are important surrogate markers of SCA that represent an increased risk of CV events [8,9]. The present study investigated the effects of MS and its components on SCA according to diabetes status in a sample of 2,560 adults who participated in baseline health examinations for a community-based cohort study.

## Methods

### Subjects

This is a cross-sectional investigation analyzing baseline data collected for a prospective cohort study. We used the data of 2,560 subjects who participated in baseline health examinations for a community-based cohort study in the Seoul area between April 2010 and November 2012. Subjects with a clinical history of CVD, cerebrovascular disease, neurological abnormalities, cerebral hemorrhage, or malignancy were excluded. The study protocol was approved by the local ethics committee of our institution, and informed consent for the procedure was obtained from each individual.

### Measurement of biochemical and clinical parameters

All blood samples were obtained after 8 hours of fasting and analyzed for glucose, triglycerides, high-density lipoprotein (HDL) cholesterol, and low-density lipoprotein (LDL) cholesterol. Height, weight, and waist circumference were measured while subjects wore light clothing and no shoes. Waist circumference was measured at the midpoint between the lower border of the rib cage and iliac crest. Body mass index (BMI) was calculated as weight (kg) ÷ height (m^2^). MS was defined as when 3 or more of the following were present: (a) abdominal obesity based on waist circumference ≥ 90 cm in males or ≥ 80 cm in females; (b) HDL cholesterol < 40 mg/dL in males or <50 mg/dL in females; (c) fasting triglycerides ≥ 150 mg/dL; (d) blood pressure ≥ 130 mmHg systolic or ≥ 85 mmHg diastolic, or on treatment; and (e) impaired fasting glucose, defined as fasting glucose ≥ 100 mg/dL, based on the American Heart Association/National Heart, Lung, and Blood Institute (AHA/NHLBI) definition [2]. Diabetes was defined as either fasting glucose ≥ 126 mg/dL, a referral diagnosis of diabetes, or antidiabetic treatment.

### Measurement of baPWV

All subjects abstained from caffeine-containing food or beverages for at least 45 minutes prior to baPWV measurement. After a subject had been resting in the supine position for at least 5 minutes in a quiet room, blood pressure and baPWV were measured using an automated waveform analyzer (Colin VP-2000, Colin Medical Instruments Corp., Komaki, Japan). Pneumatic cuffs were wrapped around both upper arms and ankles and connected to a plethysmographic sensor to determine the volume pulse waveform; the higher value of blood pressure was used for analysis. The highest value of baPWV measured on either side of each patient was used for analysis.

### Measurement of carotid IMT and plaques

Carotid IMT was measured using high-resolution B-mode ultrasonography (Acuson X300, Siemens, USA) with a transducer frequency of 13–15 MHz. Computer-assisted acquisition, processing, B-mode images storage, and calculation of IMT were performed using the Syngo Arterial Health Package (Siemens, USA). Automatic measurements from both common carotid arteries were made at the far wall of the 1-cm segment distal to the carotid bulbs. The mean value of carotid IMT was used for analysis. All carotid IMT measurements were taken at sites free of any discrete plaques. Carotid plaque was defined as the presence of focal wall thickening at least 50% greater than that of the surrounding vessel wall or as a focal region with a carotid IMT greater than 1.5 mm, protruding into the lumen and distinct from the neighboring boundary [10,11].

### Statistical analysis

Clinical and biochemical characteristics are shown according to the presence of diabetes and MS. Values are expressed as mean ± SD. Continuous variables were compared using Student’s *t*-test, and categorical variables were compared using the *χ*^2^ test. Differences in baPWV and carotid IMT with respect to each MS component were tested using ANCOVA separately for individuals with and without diabetes. Correlational analysis between the number of MS components, and baPWV and carotid IMT according to diabetes status was performed using Pearson’s correlation test. Multiple regression analysis was used to evaluate the significance of confounding risk factors for baPWV and carotid IMT. Multiple logistic regression analysis was used to evaluate the significant risk factors for carotid plaques. SPSS version 18 (SPSS Inc., Chicago, IL, USA) was used for all statistical analyses. All statistical tests were 2-tailed, and p < 0.05 was considered significant.

## Results

### Clinical characteristics

The clinical characteristics of the 2,560 participants (60 ± 8 years, 33% men) in this study are shown in Table 1. This study included 2,149 non-diabetics (84%) and 411 diabetics (16%). Both non-diabetics and diabetics were classified into 2 subgroups based on the presence of MS. The prevalence of AHA/NHLBI-defined MS in the present study was 40%; 667 non-diabetics (31%) and 285 diabetics (69%) were classified as having MS, respectively.

**Table 1:** Clinical characteristics of the study subjects

**Characteristics**	**No diabetes**			**p**	**Type 2 diabetes**			**p**
	**Total (n = 2149)**	**No metabolic syndrome (n = 1482)**	**Metabolic syndrome (n = 667)**		**Total (n = 411)**	**No metabolic syndrome (n = 126)**	**Metabolic syndrome (n = 285)**	
Age (years)	60 ± 8	59 ± 8	62 ± 7	<0.001	63 ± 8 *	63 ± 7	63 ± 8	0.354
Men, n (%)	638 (30%)	428 (29%)	210 (32%)	0.222	204 (50%) *	68 (54%)	136 (48%)	0.243
Waist circumference (cm)	83 ± 8	81 ± 8	88 ± 7	<0.001	88 ± 8 *	81 ± 7	90 ± 8	<0.001
Body mass index (kg/m^2^)	24.7 ± 2.9	24.0 ± 2.7	26.3 ± 2.8	<0.001	25.5 ± 3.2 *	23.3 ± 2.2	26.5 ± 3.1	<0.001
Smoking, n (%)	521 (24%)	336 (23)	185 (28%)	0.011	174 (42%) *	52 (41%)	122 (43%)	0.771
Heart rate (bpm)	66 ± 9	66 ± 9	67 ± 10	0.080	70 ± 10 *	69 ± 10	70 ± 10	0.304
SBP (mmHg)	122 ± 15	119 ± 14	128 ± 15	<0.001	128 ± 15 *	124 ± 15	130 ± 15	<0.001
DBP (mmHg)	74 ± 10	72 ± 9	77 ± 10	<0.001	76 ± 10 *	73 ± 9	77 ± 10	<0.001
Antihypertensive drugs, n (%)	843 (39%)	416 (28%)	427 (64%)	<0.001	251 (61%) *	46 (37%)	205 (72%)	<0.001
Total cholesterol (mg/dL)	202 ± 35	202 ± 35	200 ± 35	0.379	187 ± 39 *	185 ± 34	187 ± 41	0.493
Triglyceride (mg/dL)	126 ± 67	105 ± 47	173 ± 80	<0.001	144 ± 85 *	96 ± 34	165 ± 92	<0.001
HDL cholesterol (mg/dL)	55 ± 15	59 ± 14	46 ± 12	<0.001	50 ± 13 *	57 ± 11	47 ± 13	<0.001
LDL cholesterol (mg/dL)	124 ± 32	124 ± 32	124 ± 33	0.929	110 ± 35 *	109 ± 31	111 ± 36	0.524
Lipid lowering drugs, n (%)	571 (27%)	384 (26%)	187 (28%)	0.302	149 (36%) *	45 (36%)	104 (37%)	0.880
Creatinine (mg/dL)	0.78 ± 0.18	0.78 ± 0.18	0.79 ± 0.18	0.029	0.82 ± 0.22 *	0.81 ± 0.24	0.83 ± 0.21	0.464
FBS (mg/dL)	95 ± 9	93 ± 8	100 ± 10	<0.001	132 ± 33 *	128 ± 36	133 ± 32	0.187
Antidiabetic treatments, n (%)	-	-	-	-	347 (84%) *	112 (89%)	235 (83%)	0.097
Duration of diabetes (months)	-	-	-	-	117 ± 91	131 ± 102	110 ± 85	0.058
baPWV (cm/s)	1476 ± 250	1438 ± 230	1562 ± 271	<0.001	1641 ± 284 *	1609 ± 273	1655 ± 288	0.133
Carotid IMT (mm)	0.76 ± 0.20	0.73 ± 0.19	0.81 ± 0.22	<0.001	0.84 ± 0.26 *	0.83 ± 0.25	0.84 ± 0.26	0.773
Carotid plaques, n (%)	453 (21%)	273 (18%)	180 (27%)	<0.001	148 (36%) *	51 (41%)	97 (34%)	0.222

Data are expressed as n (%) or mean ± SD. *baPWV*, brachial–ankle pulse wave velocity; *DBP*, diastolic blood pressure; *FBS*, fasting blood sugar; *HDL*, high-density lipoprotein; *IMT*, intima–medial thickness; *LDL*, low-density lipoprotein; *SBP*, systolic blood pressure. *p < 0.001 vs. no diabetes.

### Differences in SCA parameters according to the presence of MS and diabetes

**Figure 1:**
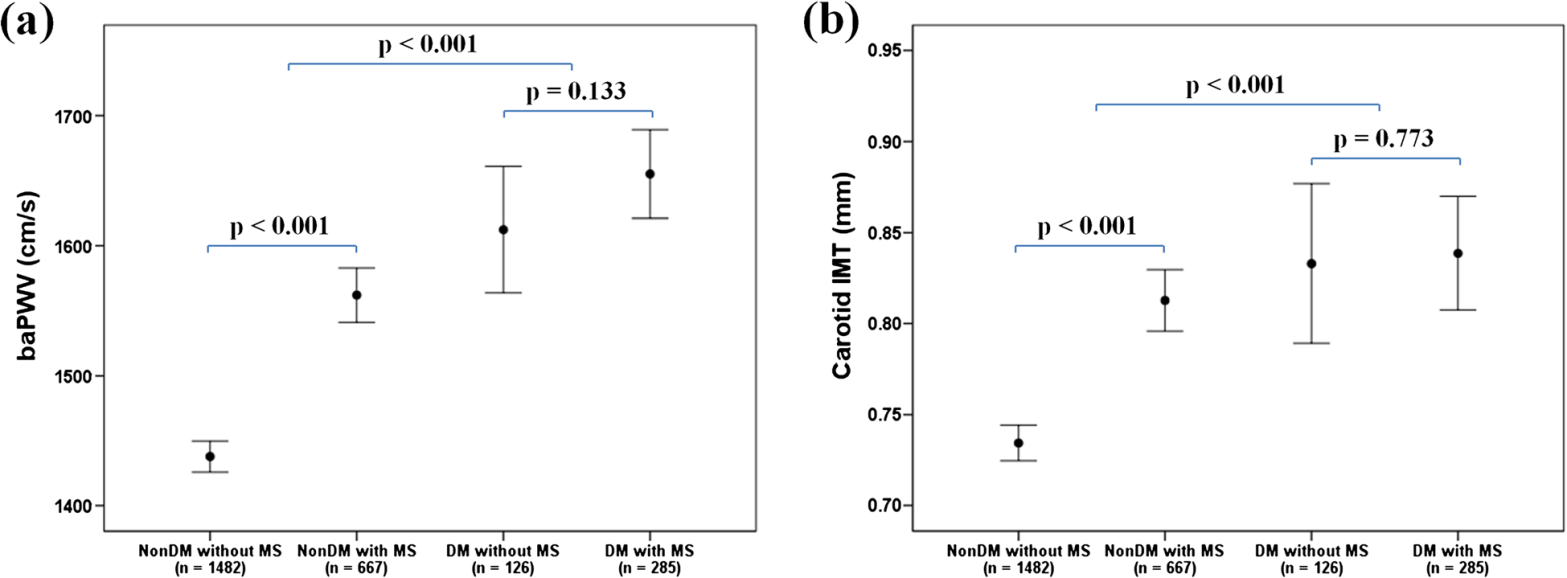
**Comparison of SCA parameters according to the presence of MS and diabetes.** (**a**) baPWV, (**b**) carotid IMT. DM, diabetes mellitus.

The mean baPWV, carotid IMT, and prevalence of carotid plaques were significantly higher in diabetics than non-diabetics (baPWV: 1641 ± 284 vs. 1476 ± 250 cm/s, p < 0.001; carotid IMT: 0.84 ± 0.26 vs. 0.76 ± 0.20 mm, p < 0.001; carotid plaques: 36vs.21%, p < 0.001). Subjects with MS had significantly higher baPWV and carotid IMT as well as more plaques than those without MS among only non-diabetics (baPWV: 1562 ± 271 vs. 1438 ± 230 cm/s, p < 0.001; carotid IMT: 0.81 ± 0.22 vs. 0.73 ± 0.19 mm, p < 0.001; carotid plaques: 27 vs. 18%, p < 0.001) (Table 1 and Figure 1). Data related to comparison of SCA parameters between 4 groups are provided in Additional file 1: Table S1.

### Impact of individual MS component on SCA parameters according to diabetes status

After adjusting for age, gender, smoking status, LDL, and BMI, MS components including increased blood pressure, triglycerides and fasting glucose as well as decreased HDL were significantly associated with higher baPWV in non-diabetics. MS components including increased waist circumference and blood pressure, and decreased HDL were significantly associated with higher carotid IMT in non-diabetics. Increased blood pressure was significantly associated with a higher risk of carotid plaques and the exacerbation of all parameters of SCA in non-diabetics. However, increased blood pressure was only significantly associated with higher baPWV among all MS components and no components of MS affected other SCA parameters in diabetics (Table 2).

**Table 2:** Parameters of SCA according to the presence of MS components in both non-diabetics and diabetics

**Characteristic**	**n**	**baPWV (cm/s)**	**Carotid IMT (mm)**	**Carotid plaques OR (95% CI)**
**No diabetes**				
Increased waist circumference				
No	1020	1471 ± 8	0.74 ± 0.01	1.00
Yes	1129	1482 ± 7	0.78 ± 0.01 *	0.96 (0.73–1.27)
Increased blood pressure				
No	1008	1393 ± 7	0.73 ± 0.01	1.00
Yes	1131	1550 ± 6 *	0.78 ± 0.01 *	2.03 (1.59–2.59) *
Increased triglycerides				
No	1584	1458 ± 5	0.76 ± 0.01	1.00
Yes	565	1528 ± 9 *	0.76 ± 0.01	0.97 (0.76–1.24)
Decreased HDL				
No	1504	1467 ± 6	0.75 ± 0.01	1.00
Yes	645	1497 ± 9 ^†^	0.78 ± 0.01 *	1.15 (0.92–1.45)
Increased fasting glucose				
No	1523	1467 ± 6	0.76 ± 0.01	1.00
Yes	626	1500 ± 9 ^†^	0.76 ± 0.01	1.10 (0.87–1.40)
**Type 2 diabetes**				
Increased waist circumference				
No	145	1657 ± 26	0.84 ± 0.02	1.00
Yes	266	1632 ± 18	0.84 ± 0.02	0.81 (0.47–1.41)
Increased blood pressure				
No	95	1534 ± 27	0.81 ± 0.03	1.00
Yes	316	1673 ± 14 *	0.84 ± 0.01	1.16 (0.70–1.95)
Increased triglycerides				
No	282	1637 ± 16	0.83 ± 0.02	1.00
Yes	129	1650 ± 23	0.85 ± 0.02	1.04 (0.65–1.65)
Decreased HDL				
No	264	1649 ± 16	0.84 ± 0.02	1.00
Yes	147	1627 ± 22	0.84 ± 0.02	1.06 (0.68–1.66)

Data are expressed as mean ± SD. All models are adjusted for age, gender, smoking status, LDL, and BMI. *baPWV*, brachial–ankle pulse wave velocity; *HDL*, high-density lipoprotein; *IMT*, intima–medial thickness; *LDL*, low-density lipoprotein. *p < 0.001; ^†^p < 0.01; ^‡^p < 0.05.

### Relationship between the number of MS components and SCA parameters according to diabetes status

**Figure 2:**
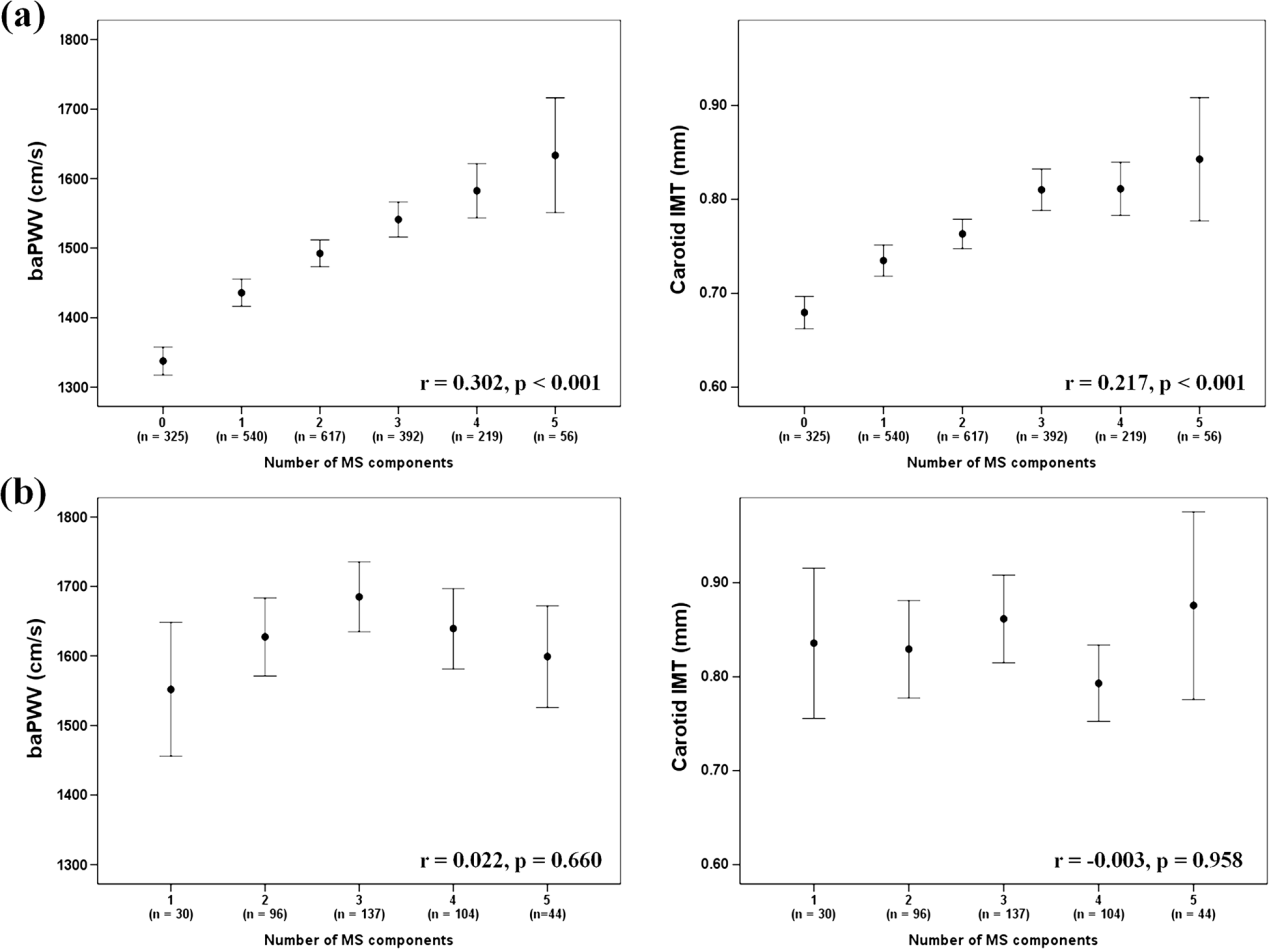
**Correlations between the number of MS components, and baPWV and carotid IMT.** (**a**) Non-diabetics, (**b**) diabetics.

The number of MS components was significantly correlated with baPWV and carotid IMT in non-diabetics (baPWV: r = 0.302, p < 0.001; carotid IMT: r = 0.217, p < 0.001). However, it was not significantly correlated with either in diabetics (baPWV: r = 0.022, p = 0.660; carotid IMT: r = −0.003, p = 0.958) (Figure 2).

### Impact of MS and diabetes on SCA parameters

Multiple linear regression analysis for baPWV and carotid IMT was performed using age, gender, smoking status, BMI, LDL, MS, and diabetes (Table 3). The results showed that age (β = 14.516, p < 0.001), male sex (β = 52.753, p < 0.001), MS (β = 87.450, p < 0.001), and diabetes (β = 87.408, p < 0.001) were significantly associated with baPWV. Meanwhile, age (β = 0.010, p < 0.001), male sex (β = 0.057, p = 0.001), LDL cholesterol (β = 0.001, p < 0.001), MS (β = 0.042, p < 0.001), and diabetes (β = 0.031, p = 0.005) were significantly associated with carotid IMT. Multiple logistic regression analysis was performed for carotid plaques using the same covariates (Table 3). The results showed that age (odds ratio [OR], 1.08; 95% confidence interval [CI], 1.07–1.10; p < 0.001), male sex (OR, 1.36; 95% CI, 1.00–1.85; p = 0.050), MS (OR, 1.26; 95% CI, 1.01–1.57; p = 0.041), and diabetes (OR, 1.50; 95% CI, 1.17–1.93; p = 0.002) were significantly associated with the prevalence of carotid plaques.

**Table 3:** Multiple linear logistic regression analysis of the determinants of baPWV, and carotid IMT and plaques

	**baPWV**			**Carotid IMT**			**Carotid plaques**	
	**β**	**SE**	**p**	**β**	**SE**	**p**	**OR (95% CI)**	**p**
Age	14.516	0.569	<0.001	0.010	0.001	<0.001	1.08 (1.07–1.10)	<0.001
Sex (male)	52.753	14.365	<0.001	0.057	0.013	<0.001	1.36 (1.00–1.85)	0.050
Smoking	−8.674	15.121	0.571	−0.013	0.013	0.307	1.37 (0.99–1.88)	0.054
BMI	2.933	1.603	0.067	0.002	0.001	0.199	0.99 (0.96–1.03)	0.576
LDL	0.257	0.136	0.059	0.001	0.001	<0.001	1.00 (0.99–1.00)	0.786
MS	87.450	10.394	<0.001	0.042	0.009	<0.001	1.26 (1.01–1.57)	0.041
Diabetes	87.408	12.802	<0.001	0.031	0.011	0.005	1.50 (1.17–1.93)	0.002

*baPWV*, brachial–ankle pulse wave velocity; *BMI*, body mass index; *IMT*, intima–medial thickness; *LDL*, low-density lipoprotein; *MS*, metabolic syndrome.

## Discussion

To the best of our knowledge, the present study provides the first information on the differential impact of MS on atherosclerotic changes according to diabetes status. The AHA/NHLBI criteria were chosen because they are easy to apply to clinical and epidemiological studies, clearly define each MS component regarding medication status, and follow the current criteria of impaired fasting glucose [12].

Several previous studies assessed the association between MS and atherosclerosis. In the Baltimore Longitudinal Study of Aging (BLSA), Scuteri et al. [13] found that subjects with MS have significantly greater carotid IMT and stiffness than subjects without MS. Nakanish et al. [14] found that clustered features of MS are closely related to the risk of increased aortic PWV in middle-aged Japanese men. However, these studies raised substantial concerns about age-associated increases in vascular stiffness and thickness. Furthermore, they did not consider that MS was not a clinical diagnosis but rather a pre-morbid condition for the development of diabetes, which is closely associated with atherosclerosis. The present study revealed that subjects with MS had greater baPWV, carotid IMT, and plaques than those without MS among non-diabetics. However, this impact of MS on SCA was not observed in subjects with establish diabetes, although both MS and diabetes were independently associated with all vascular parameters after considering risk factors. In addition, the number of MS components was significantly associated with increases in vascular stiffness and thickness in only non-diabetics. Considering the differential impact of MS on SCA according to the presence of diabetes, it might be important to identify the presence of MS in non-diabetic individuals. However, a concurrent diagnosis of MS in individuals with established diabetes might be of little value for the risk stratification of CVD.

The present study identified different impacts of the individual components of MS on SCA, including vascular stiffness and thickness, according to diabetes status. Vascular stiffness reflected by baPWV was influenced by several MS components in non-diabetics, including increased blood pressure, triglyceride, and fasting glucose as well as decreased HDL. However, only increased blood pressure affected vascular stiffness in diabetics. This might be closely associated with the concrete relationship between baPWV and blood pressure irrespective of diabetes status. On the contrary, vascular thickness reflected in carotid IMT and plaque was influenced by MS components, including increased waist circumference and blood pressure, and decreased HDL; however, no MS components significantly affected vascular thickness in diabetics. These results suggest that the progression of atherosclerosis might be directly dependent upon hyperglycemia in patients with established diabetes status [15,16] but might be influenced by multiple CV risk factors, especially the component of increased blood pressure [17], in patients with a status of MS without diabetes.

MS has recently been promoted as a means of identifying the risk of diabetes development. Gupta et al. [18] found that both impaired fasting glucose and MS can predict the risk of new-onset diabetes and that MS is a better predictor of the risk of new-onset diabetes in hypertensive patients. In contrast, Stern et al. [19] reported that MS is inferior to the Framingham Risk Score, an established predictive model for either type 2 diabetes or CVD. In the present study, although we did not analyze the significance of MS as a predictor of type 2 diabetes development, diabetics had a significantly greater risk of SCA than non-diabetics, independent of MS status. These results suggest that diabetes strongly influences atherosclerosis independent of MS and highlight importance of identifying the new development of diabetes in non-diabetics with MS.

MS and diabetes share many common characteristics; 65–85% of diabetic individuals have MS [20-22]. However, only a few studies have examined the effect of the combination of MS and diabetes on the risk of CVD, and their results are inconsistent. Malik et al. [23] showed that individuals with MS but not diabetes have increased risks of CHD and CVD, and that diabetes predicts CHD, CVD, and overall mortality. Alexander et al. [21] reported that the prevalence of CHD is substantially higher in subjects with both diabetes and MS than in those with only diabetes. Tong et al. [22] showed that the presence of MS is associated with an increased risk of CHD in Chinese individuals with diabetes. On the contrary, Church et al. [24] reported that the presence of diabetes is associated with a 3-fold greater CVD mortality risk and that MS status does not affect this risk in men from the Aerobics Center Longitudinal Study (ACLS). In addition, while MS and diabetes confer an increased risk of CVD, recent evidence suggests that subjects with these conditions have a wide range of increased risks [25-27]. Malik et al. [26] reported that subjects with MS or diabetes have low risks of CHD when carotid IMT or coronary artery calcium (CAC) is not elevated. Furthermore, they reported that CAC predicts CVD and CHD events better than carotid IMT. Wong et al. [27] reported that subjects with MS and diabetes have a greater incidence and progression of CAC than those without these conditions; moreover, progression also predicts CHD events in those with MS and diabetes. The evaluation of baPWV and carotid IMT in the present study might be insufficient to stratify the CV risk in diabetic individuals because these SCA markers were not significantly different between diabetics with and without MS. Therefore, further investigations might be required for complete CV risk stratification and should include the assessment of morphological and functional vascular damage as well as serological markers in patients with MS and diabetes.

MS is a pre-morbid condition rather than a clinical diagnosis and has been advocated as a useful clinical tool for predicting diabetes and CVD. Although a number of different definitions of MS include diabetes as a diagnostic criterion of MS, the World Health Organization (WHO) strongly recommended that the conditions of established diabetes or CVD should be excluded in the definition of MS and proposed research that justifies the inclusion of type 2 diabetes in the definition [7]. Given the current controversy over the definition of the MS, the present result that diabetes strongly influences SCA irrespective of the presence of MS is good evidence arguing against the inclusion of patients with established type 2 diabetes in the domain of MS.

This study has some limitations. First, the criteria of MS might be dependent on race and ethnicity [28]. However, the present study included only a Korean population. Second, the impact of MS on the progression of atherosclerosis might somewhat differ according to age group [29]. However, no sub-analysis of SCA according to age group was not performed because the participants of this study were relatively older. Third, a previous study reported that dynamic endurance training favorably affects most of the CV risk factors related to MS [30]. However, we did not evaluate the physical activity of participants. Fourth, there were relatively few subjects with diabetes compared to those without diabetes because our study was a community-based cohort study. Fifth, we could not eliminate the possible effects of underlying disease and medication for hypertension, dyslipidemia, and diabetes on atherosclerosis because of the observational design of this study. Finally, we did not evaluate the degree of hyperglycemic control using HbA_1_c in diabetic patients. Further prospective studies with larger sample sizes are required to address these issues.

## Conclusions

In conclusion, MS has an incremental impact on SCA in conditions without diabetes. The identification of MS and its individual components is more important for the risk stratification of CVD in non-diabetic individuals.

## Abbreviations

ACLS: Aerobics Center Longitudinal Study; AHA/NHLBI: American Heart Association/National Heart, Lung, and Blood Institute; baPWV: Brachial–ankle Pulse Wave Velocity; BLSA: Baltimore Longitudinal Study of Aging; BMI: Body Mass Index;CAC: Coronary Artery Calcium; CHD: Coronary Heart Disease; CI: Confidence Interval;CV: Cardiovascular; CVD: Cardiovascular Disease; DBP: Diastolic Blood Pressure; FBS: Fasting Blood Sugar; HDL: High-Density Lipoprotein; IMT: Intima–Medial Thickness; LDL: Low-Density Lipoprotein;MS: Metabolic Syndrome; OR: Odd Ratio; SBP: Systolic Blood Pressure; SCA: Subclinical Atherosclerosis; SD: Standard Deviation; WHO: World Health Organization.

## Competing interests

The authors declare that they have no competing interest.

## Authors’ contributions

All authors listed in the manuscript participated in the design of the study and in writing the manuscript. KW and SS performed the statistical analysis. All authors read and approved the final manuscript.

## Supplementary Material

Additional file 1: Table S1 Comparison of SCA parameters between the 4 groups. Click here for file
