# Supplementary material for: Differential impact of metabolic syndrome on subclinical atherosclerosis according to the presence of diabetes
Source: Cardiovasc Diabetol. 2013 Mar 4;12:41. doi: 10.1186/1475-2840-12-41 (PMC3599532; doi:10.1186/1475-2840-12-41)
Supplement: Additional file 1: Table S1 — Comparison of SCA parameters between the 4 groups. [file 1475-2840-12-41-S1.doc]

**Table** Comparison of SCA parameters between the 4 groups

|  | **Non-DM without MS (n** **=** **1482)** | **Non-DM with MS (n** **=** **667)** | **DM without MS (n** **=** **126)** | **DM with MS (n** **=** **285)** |
| --- | --- | --- | --- | --- |
| baPWV (cm/s) | 1438 ± 230 | 1562 ± 271 * | 1609 ± 273 * | 1655 ± 288 *^†^ |
| Carotid IMT (mm) | 0.73 ± 0.19 | 0.81 ± 0.22 * | 0.83 ± 0.25 * | 0.84 ± 0.26 * |
| Carotid plaques, n (%) | 273 (18%) | 180 (27%) * | 51 (41%) *^†^ | 97 (34%) *^†^ |

Data are expressed as n (%) or mean ± SD. Differences between groups were tested using the *χ*^2^ and ANOVA with Scheffe’s post hoc test performed if p < 0.05. *p < 0.05 vs. non-DM without MS. ^†^p < 0.05 vs. non-DM with MS. ANOVA, analysis of variance; baPWV, brachial–ankle pulse wave velocity; DM, diabetes mellitus; IMT, intima–medial thickness; MS, metabolic syndrome.
